# Artificial intelligence systems for complex decision-making in acute care medicine: a review

**DOI:** 10.1186/s13037-019-0188-2

**Published:** 2019-02-01

**Authors:** Lawrence A. Lynn

**Affiliations:** The Sleep and Breathing Research Institute, 1251 Dublin Rd, Columbus, OH 43215 USA

## Abstract

The integration of artificial intelligence (AI) into acute care brings a new source of intellectual thought to the bedside. This offers great potential for synergy between AI systems and the human intellect already delivering care. This much needed help should be embraced, if proven effective. However, there is a risk that the present role of physicians and nurses as the primary arbiters of acute care in hospitals may be overtaken by computers. While many argue that this transition is inevitable, the process of developing a formal plan to prevent the need to pass control of patient care to computers should not be further delayed.

The first step in the interdiction process is to recognize; the limitations of existing hospital protocols, why we need AI in acute care, and finally how the focus of medical decision making will change with the integration of AI based analysis. The second step is to develop a strategy for changing the focus of medical education to empower physicians to maintain oversight of AI. Physicians, nurses, and experts in the field of safe hospital communication must control the transition to AI integrated care because there is significant risk during the transition period and much of this risk is subtle, unique to the hospital environment, and outside the expertise of AI designers.

AI is needed in acute care because AI detects complex relational time-series patterns within datasets and this level of analysis transcends conventional threshold based analysis applied in hospital protocols in use today. For this reason medical education will have to change to provide healthcare workers with the ability to understand and over-read relational time pattern centered communications from AI. Medical education will need to place less emphasis on threshold decision making and a greater focus on detection, analysis, and the pathophysiologic basis of relational time patterns. This should be an early part of a medical student’s education because this is what their hospital companion (the AI) will be doing.

Effective communication between human and artificial intelligence requires a common pattern centered knowledge base. Experts in safety focused human to human communication in hospitals should lead during this transition process.

## Background

### Facing the challenge from Silicon Valley

Three thousand years ago the perceived power of the human brain to affect survival from illness was hyperbolized in Asclepius, the Greek god of medicine. Asclepius was credited with such a powerful intellect that he altered the ratio of living to dead.

While mortal physicians have never matched his success, for thousands of years patients have placed their confidence in the intellect of physicians for medical diagnosis and care. However, the present role of physicians and nurses as preeminent diagnosticians and providers of care may soon be overtaken by computers. While many argue that this transition is inevitable, physicians have not yet developed a formal plan to respond to the challenge from Silicon Valley. This is a momentous time and the process of developing a formal plan to prevent the need to pass the Caduceus should not be further delayed.

The first step in the prevention of loss of the position of physicians and nurses as preeminent overseers of hospital care is to understand the present limitations of medical diagnostics in the acute care environment and in medical education which have driven the need for AI integration.

### The present state of acute care decision making

A computer programmer examining the present threshold based hospital protocols with the intent to develop algorithms for managing care might quickly conclude that automation of acute care diagnostics and treatment would be easy to implement. The reason for this is that present hospital protocols are based on twentieth century threshold decision making [1] and are generally quite simple. In an example, it might appear to a computer programmer that all that is required to diagnose and treat sepsis is an indication that infection is suspected, a simple threshold breach detection algorithm [2] and a branching set of treatment rules.

However expert clinicians know that these simple protocols are not indicative of the true levels of acute care complexity [3, 4]. The randomized controlled trials (RCT) which use the threshold rules applied in hospital protocols as unified standards for an entire population are subject to marked heterogeneous treatment effects (HTE) [5]. Such trials provide evidence of the average treatment effect on the group under test as a whole but not whether the treatment used in the RCT will be beneficial or harmful to the instant patient under care. It therefore logically follows that no protocol, no matter how well supported by RCT, can be applied without expert oversight provided by either by a human or AI to protect patients from harm.

For this reason designers of automated systems must recognize that the true complexity of diagnosis and management of adverse conditions, such as sepsis, resides in the portion of diagnosis and care provided by nuanced expert oversight which is difficult to study and reproduce. If the patient is worsening despite adherence to the standard protocol, a physician with naïve trust in RCT may adhere to the guidelines thinking she must stay the course. In the alternative the expert physician knows to detect and track worsening, knows the potential for HTE, and modifies care off protocol if necessary. In addition to responding to worsening, expert clinical nuance is also applied to change diagnosis and/or alter the care in the presence of an unusual presentation, rare cases, and less common mixes of overlapping diseases.

**Fig. 1:**
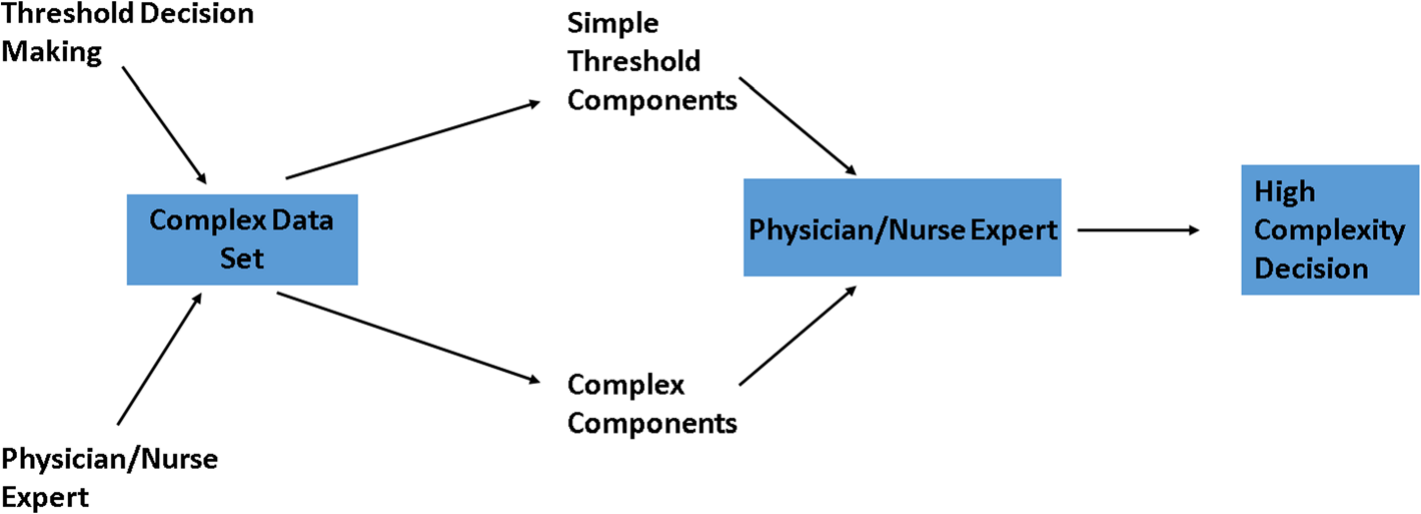
The present state of nuanced threshold decision making. Threshold protocols are simple and transparent so nuanced care with complex decisions outside the protocols can be provided by the nurse/physician when needed

Figure 1 illustrates the present state of hospital care based on uniform protocols with physicians and nurses operating as expert overseers, protecting patients from the hazards of oversimplified threshold based decisions. Physicians and nurses can provide oversight because the decision process of the protocols are transparent so, using their own intellect, they are able to modify the diagnosis and treatment as needed in real-time.

## The integration of artificial intelligence into acute care

### AI and the acute care environment

The future of medical AI extends far beyond the interpretation of medical images, pathology slides and radiographs. AI is being developed to detect critical, highly complex and time dependent conditions such as adverse drug reactions and sepsis [6, 7] in acute care environments where timely nuanced communication is pivotal. It is likely that within 5 years, AI based protocols will replace many of the threshold based protocols which are presently providing acute care diagnostic and treatment decisions.

One advantage of AI is that it can analyze more relationally complex portions of a patient’s dataset. However, a major disadvantage is that the complex decision processing of the AI may be substantially opaque if not designed to provide transparency and nuanced communication. Effective communication from an AI must not be inferior to communication from a human.

However the need for detailed real-time communication is even more acute when the adverse clinical conditions under investigation and care are highly complex and rapidly progressing critical conditions. For this reason, the introduction of AI into the acute care environment should be preceded by detailed consideration of how AI, if not properly implemented, may cause harm by adversely affecting communication as well as the role of the physician and nurse as expert overseers of care.

### The hazards of black-box artificial intelligence in acute care

In the worst case, complex analytics may be provided by AI without disclosure of the data used to make the decision or the analytic processes applied. This is often called “black-box AI”. This approach is reasonable in acute care only if expert human oversight will not be beneficial even if the patient is worsening. One example of inadequate black box AI would be the application of an AI based protocol which, after an opaque analysis of a complex data set, provides only an output stating “Sepsis detected--severity score 20”. This limited output would constitute a communication error by human standards because it does not communicate the relational time patterns (RTP) of laboratory tests and vitals detected, the other factors considered, how the patterns were combined, which component patterns are rapidly worsening, and how the overall state of the sepsis pattern has progressed over time for example in relation to treatment or to a potentially inciting event such as a surgery. This level of communication would be required if the detection was performed by a human expert so the AI must deliver equivalent or better communication.

While black box AI is clearly unacceptable in acute care, the standards for AI transparency and for timely communication by AI of the details of its decision process have not been determined. Yet it is clear that AI should meet the minimum standards of detail required for physician to physician handoff.

**Fig. 2:**
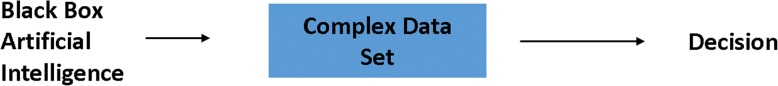
The bedside state with “Black Box AI” based decision making. Complex decisions made by AI without transparency or detailed timely communication of the factors considered and how these factors were combined to render decision process. If the patient worsens under the care of the AI the handoff to the physician will be blind

Figure 2 shows that the use of Black Box AI (which has not been programmed to communicate the nuanced details of the decision process) renders a state wherein the AI stands alone. Oversight by the expert is not facilitated because she cannot see the pattern combinations detected by the AI.

### Medical diagnostic analysis is more complex than in many other environments

One problem with AI use in acute care which may not be readily apparent to those with a limited understanding of the medical domain is the common delay between the decision and the result which occurs responsive to the decision. Often it is not clear that a medical decision was wrong for a given patient’s condition until many days later when complications, recovery failure, or worsening occurs. Contrast this to AI based autonomous driving. Here, a human in the car can rapidly intervene because the correctness of the decisions made by the AI are generally immediately transparent to the overseeing driver. The immediacy of feedback simplifies the training processes of AI based autonomous driving and allows the use of black box AI because the decision process is largely irrelevant and can be opaque to the driver who is only concerned with the result which is promptly apparent. By comparison, in medical care, where the correctness of the decision is often not immediately obvious, the clinician responsible for the patient cannot simply wait with faith to see the outcome. Rather the clinician needs to be able to see the decision making process itself in fine relational detail and in real-time (before the outcome) to be sure it applies to the complexities and comorbidities of the instant patient under care. Here we can easily see the need for a new focus of medical education because bedside transparency of AI is not useful if the physician is not trained to be able to interpret the RTP detected by the AI.

### The handoff from an artificial intelligence system to a physician

The addition of AI brings new communication challenges to a complex hospital environment already associated with a high error rate. Communication errors, including those associated with patient handoffs between multiple humans, have been cited by the Joint Commission as the root cause of up to 60% of adverse events [8, 9]. Yet any action which moves care from one caregiver to another is a handoff. This is true even if a computer is the diagnostician and is now handing off the care to a human who is expected to deliver treatment. Guidelines for handoffs involving AI should be prioritized to assure they are ready when AI is integrated into hospital care.

In addition the danger of the potential delay between decision and result will likely be most evident when a patient managed by Black Box AI fails to recover. The clinician will want to know if a mistake was made in AI based diagnoses or in the therapeutic choices made or both. Here the need for real-time and detailed communication is evident. The clinician must know which components of the data set comprised the basis for the AI decision and what part of the decision process might be wrong. The answers to these pivotal questions will not be evident when detailed real time communication is lacking and the physician is blind to processing. Again this shows the need for refocused education since transparency and disclosure of RTP detected by the AI are only useful to the physician if she is trained to interpret them.

Fortunately there is already a major body of literature directed toward the study of factors which induce error during human to human communication in the patient care environment. Under these guidelines, a physician handing off care to another must provide detailed communication which explains the time patterns or threshold breaches identified in the data, the diagnostic considerations, and the state and rational for various therapies applied or under consideration. With those lessons learned, it follows that an AI system which is entrusted with critical clinical decisions, should be considered a human equivalent for the purpose of defining AI communication protocols and standards.

### Artificial intelligence as a cause of clinical dependency

The second problem which may develop over time in the acute care environment comprises intellectual dependency on AI, especially AI which lacks detailed communication capability. The lack of detailed communication renders the state presented in Fig. 2 which will reduce the perceived need for nurses and physicians to learn complex pathophysiology or to intellectually engage the complexity of care. As the perceived need to learn complex pathophysiology diminishes, so too does the competency of the physician or nurse.

Perhaps there will come a time when AI is so highly effective that loss of the quality of the oversight function of nurses and physicians will not be a concern. However, over the next few decades we must focus on the transition state. This is the present state of automated driving, where the performance of AI must still be evaluated and supervised by a human in real time.

### The need for a new focus of medical education

There is little doubt that there will be a diminishing role for the portions of traditional medical education which are focused on simple rules and threshold decision making. The simple threshold rules most students have been taught, for example the threshold criteria used to diagnose sepsis, will be abandoned because they are based on data fragments and the AI does not need to keep it simple. Also, physicians will not be able to rely on those simple threshold rules to over read the outputs of the AI because the weakness of those rules is one of the reasons AI is being introduced.

With the emergence of AI, simple threshold based diagnostic and treatment rules will be replaced by AI based protocols which will be much more complex and will include RTP analysis. Fortunately the teaching of threshold rules, which became popular in the late twentieth century, was always an oversimplification so the abandonment of this aspect of medical education will not be much of a loss.

However, if proper steps are not taken soon, the effect of AI on medical education has the potential to cause a much deeper cut. Indeed, it has been suggested that, in the age of AI, physicians will not need to be as intelligent as is required today. Instead, it is argued, the focus of education will be on empathy, comfort, counseling, and end of life care.

Nascent resignation to intellectual subservience to AI is driven, at least in part, by the complexity of the processing performed by the computer. The decision processing of the AI is not easy to teach and it will not be possible for many medical professionals to determine, at the bedside, the integrity of the processing itself. However that does not mean that the loss of the oversight role for the physician and nurse is inevitable. For healthcare workers to maintain their pivotal oversight role they must prepare themselves to cognitively engage the same data, the RTP and the same relationships which are detected and processed by the AI. Just as importantly they must demand that AI for acute care is configured to provide real-time communication of the analysis performed in a format which can be over read by clinicians at the bedside so that physicians can protect their patients from treatment failure due to HTE and the AI and physicians can learn together. If medical educators and physicians do not go forward with both of these steps, they will become intellectually subservient because they will not be able to oversee the integrity of decisions made by the AI.

The over reading function of the physician or nurse will be facilitated by AI driven cognitive support which presents the detected RTP and the data from which the patterns were detected to the user in an organized way. When this cognitive support is provided, the over reading of such patterns is well within the capability of physicians and nurses trained to understand them but this will require agreement on terminology.

### Time-series patterns, the “integers of physiology”

One way to advance ones understanding of biologic time patterns is to start with the most basic pattern pieces, the pattern building blocks of the biological data. Note, we are starting with pattern building blocks of the biologic data not with the biologic data itself. In other words we are going to consider fundamental time patterns of data which are reliably present regardless of the biologic process being considered. One might call these basic time patterns the “integers of physiology” as they are quite simple and universally present in all biologic time series.

Using the basic integers to build a complex pattern means that the complex patterns can be disassembled (factored) into its component parts including the components which define the onset of the pattern. This assures that the variations of complex patterns can be understood, quantified in reliably definable terms.

To understand, and communicate with, and over read time pattern based outputs of advanced medical AI, it is important to begin with a common terminology describing these fundamental integers of physiology. After this we will see how physiologic and pathophysiologic time patterns fit together as we build outward from there.

Fortunately there are only 5 primary fundamental time pattern types. This makes the terminology and the patterns easy to learn. Of course this is only one proposed terminology as no standards have been developed.

These are:

1. **Perturbation-** (a rise or fall away from the phenotypic or baseline range)
2. **Recovery** (a rise or fall from a perturbation back toward baseline)
3. **Reciprocation** (a perturbation followed by its recovery)
4. **Distortion** (a combination of perturbations induced by a common force such as a drug or invading organism)
5. **Recovery from a Distortion** (a combination of recoveries from the perturbations which comprise the distortion)

Using images of time patterns as the fundamental communication tool between AI and humans allows both the AI and the physician to succinctly communicate complexity while speaking a common language. Both still having the ability to factor these complex images into their component parts for deeper analysis to learn together and over read each other’s work.

### Biologic time patterns are relational time patterns

Unless only one dataset from a single point in time is available (which is almost never the case), the patterns detected by the AI are relational time patterns (RTP) of sequential lab values, vitals, pharmaceutical doses, and test results in relation to other things such as image results, other diagnoses, sex, age, etc. Relational time patterns (RTP) are the next level up (from simple time patterns) of pattern complexity. RTP are comprised of two or more time patterns occurring in temporal relation to each other. Since RTP are built from time pattern “integers” their composition can be widely varied and quite complex depending on how many time patterns are included and of what type. The number of potential time pattern combinations (and therefore RTP) is profound. One example of a complex RTP common in a phenotype of sepsis is comprised of; a fall in absolute neutrophil count, a rise in bands, a fall in platelets, a fall in bicarbonate and a rise in lactate. Together they comprise part of the image of a distortion induced by sepsis.

The acceptance of the view that thresholds can be remembered but RTP are too complex, to numerous and to varied in type to remember would thead to rapid decline in the role of physicians in the management of complex patients and loss of protection provided by physician oversight. There is no alternative to learning these patterns short of giving up the rod of Asclepius to the machines. However, here the computer can assist by presenting the time data in a unified picture so that the patterns are identifiable by the trained eye in the picture rendering the process of relational time pattern detection by physicians and associated AI oversight much easier.

### Seeing the data of a patient as a factorable time matrix

To understand any machine (including a biologic one), and to detect its failures, one must first construct the normal relationships of its fundamental parts. The fundamental parts of a human are not its appendages or organs but rather humans are comprised of a massive set of biologic particle densities. Examples of biologic particles include ions, cells, cell fragments, and molecules. The venous platelet count, potassium concentration, an antibody concentration, and the SPO2 or etCO2 are all examples of biologic particle densities or measurement surrogates for those densities. Like the parts of a machine, these densities also are held in a highly organized baseline operational state and change over time in a relational way within that baseline operational state.

**Fig. 3:**
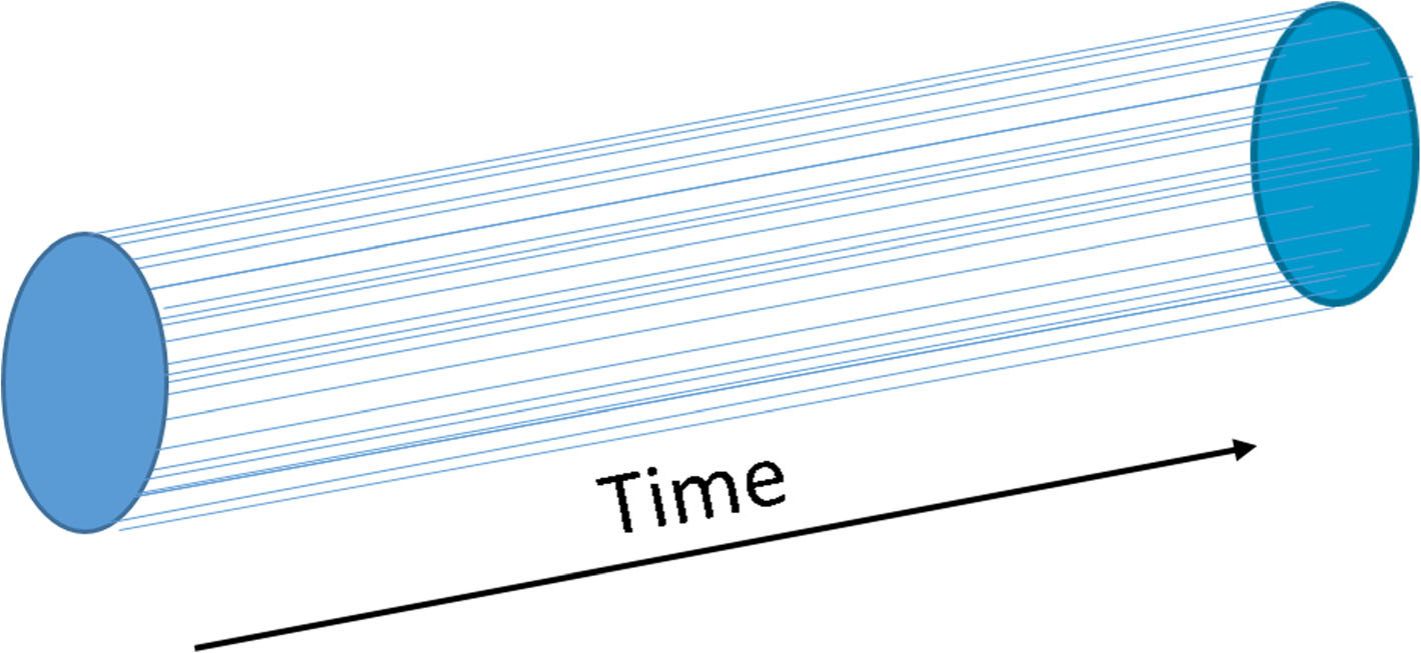
A Healthy Human Time Matrix (HTM). Humans are comprised of a matrix of biologic particle densities (e.g. bicarb, sodium, neutrophils), and forces which project along a time axis in a dynamically relational configuration. As represented in the time domain, the HTM is comprised of individual time series (waves) in specific relation to the phenotypic range and to each other (events, forces, lab values, vitals, drugs, etc.)

A human, viewed as a machine, is actually an aggregation of biologic particle densities which are held, in health, in the baseline operational state over time (Fig. 3). As with the machine, one way to cognitively engage a unified patient dataset is by defining a human time matrix model as a unified aggregation of the fundamental time patterns. With this approach it is possible to perceive an entire patient dataset as a single image which grows over the patient’s life time.

When a sufficient new force, such as a medication or invading organism, acts on the matrix, the force often causes at least one “perturbation” generated away from the phenotypic range. When the force is removed or a countermeasure is applied, a “recovery” generally occurs in the opposite direction of the perturbation. Perturbations and recoveries are vectors with features of duration, slope, magnitude, acceleration, percent change, etc. The combination of the perturbation and its recovery is a reciprocation and these can be complete (with a full recovery) or incomplete when there is recovery failure.

**Fig. 4:**
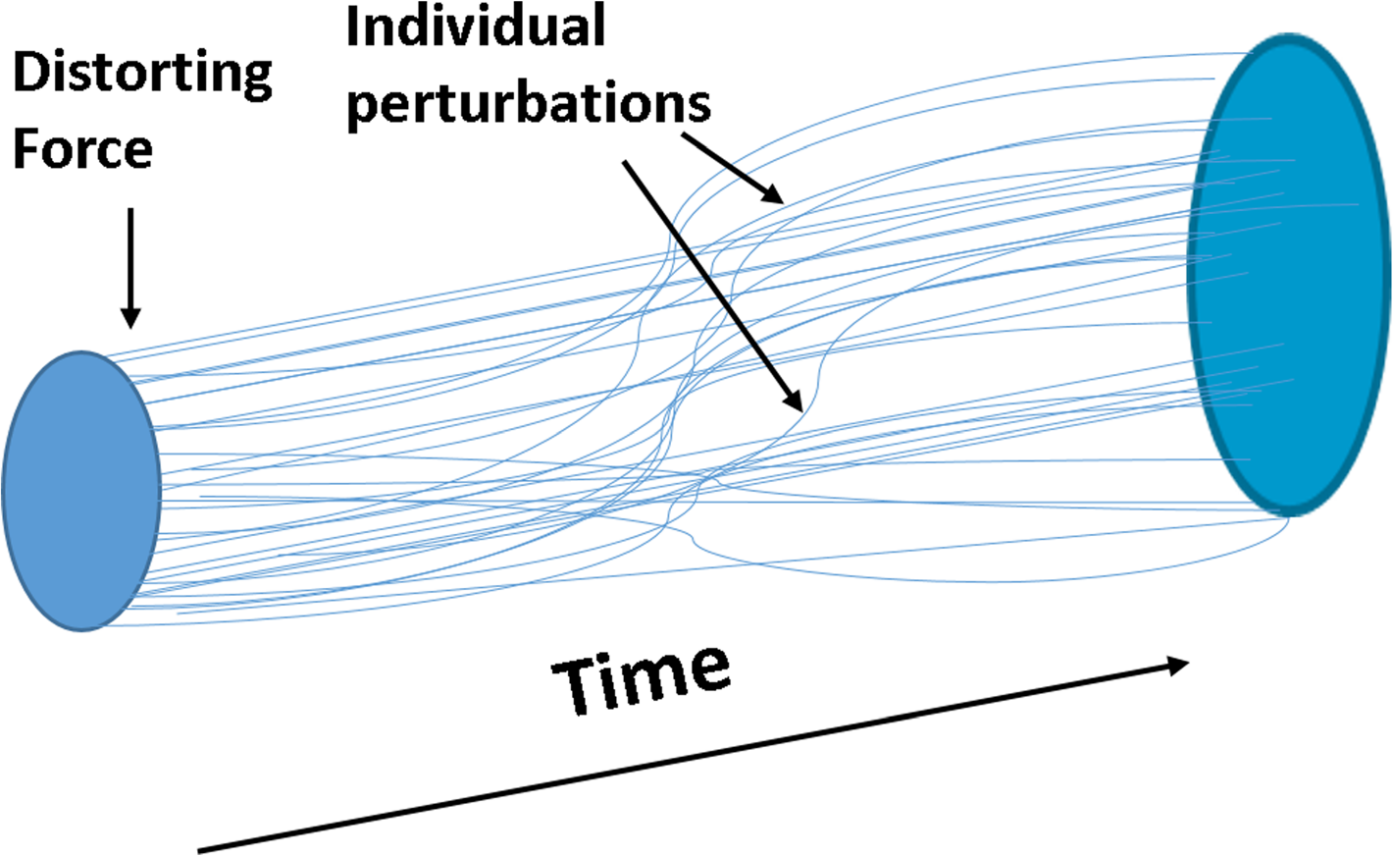
A Distortion of the Human Time Matrix. When an internal or external distortion force (e.g. infection, a drug, a pathogenic molecule) is applied to the matrix, a set of perturbations occur producing a “distortion” of the HTM

When a grouping of perturbations caused by a force occurs, this can be seen as a “distortion of the matrix” (Fig. 4). Distortions may have the temporal characteristics of onset, worsening and recovery. A distortions can be factored into its component parts and this factoring can be used by AI or the physician to track back to the earlies perturbation of the distortion when, for example, seeking the force which caused the distortion.

Figure 4 illustrates that the distortion force (e.g. infection, a drug, a pathogenic molecule) precedes the individual perturbations which form the distortion. Some perturbations occur early after the onset of the force and some occur late. The types, timing and relational patterns of these perturbations defines the time dimensioned conformation of the distortion.

Distortions produced by different adverse conditions have different conformations which change over time as severity worsens or recovery evolves. Complex conditions such as sepsis may be associated with several phenotypes of distortions.

One way to think about different phenotypes of distortions of a single condition, such as sepsis, is to consider as analogous, sequential images of different groups (image phenotypes) of pneumonia. One image group is that of lobar pneumonia, another bronchopneumonia and a third would be the scattered infiltrates and cavitation often associated with MRSA pneumonia. Although all are pneumonia the images from one group may not be very similar to the images of another and the time patterns of worsening may also be quite dissimilar. Yet, *within* each of these groups the images generally have quite similar time pattern characteristics. The same is true of groups (phenotypes) of distortions of the time matrix for a complex clinical condition.

While the oversight of AI may sound complex, it is not if the data are properly formatted and presented. By modifying medical education and turning the education focus away from simple threshold rules to the recognition of the RTP which AI systems will be detecting, physicians and nurses will be able to continue their pivotal role as overseers of care in the age of AI.

### Summary of considerations

Present acute care protocols based by twentieth century threshold decision making are inadequate and AI integration into acute care offers the potential to improve care. Yet, there is a risk that the present role of physicians and nurses as the primary arbiters of acute care in hospitals may be overtaken by computers. While many argue that this transition is inevitable, the process of developing a formal plan to prevent the need to pass control of patient care in the acute care environment to computers should not be further delayed.

The addition of AI as a new bedside intellectual source brings new communication challenges to an environment already associated with a high error rate. When a patient managed under the decision process of AI worsens, at some point the AI will need to “handoff” the patient to a physician. Without transparency this will be a blind handoff. For this reason, any AI system which determines clinical care, must be programmed to provide effective and detailed communication with clinicians as any action which moves care from one caregiver to another is a handoff. This is true even if a computer is the diagnostician and is now handing off the care to a human who is expected to deliver treatment. The patient safety requirements for handoffs, which include full transparency, must be maintained.

Physicians and nurses must control the transition to AI integrated acute care because there is significant risk during the transition period and much of this risk is subtle, unique to the medical environment, and outside the expertise of AI designers.

AI systems detect and analyze relational time patterns and oversight of AI using conventional threshold decision making will not be effective. For this reason, medical education will have to change to provide healthcare workers with the ability to understand AI communications. An increased focus on teaching clinical time pattern recognition is required. Students should be taught in early phases of their medical education to factor complex relational time patterns and to think in relational time pattern terms.

Effective communication between human and artificial intelligence requires a new relational time pattern centered knowledge base and terminology. This terminology must support detailed and nuanced communication with AI. Experts in safety focused human to human communication in hospitals should lead during this transition process.

It is the functional hybridization of human and artificial intelligence at the bedside, which offers the greatest hope for safely revolutionizing medical care over the next decade. To achieve this lofty goal early collaboration by experts from many diverse fields of study is required.

## Conclusion

The introduction of an artificial intellect to the bedside has the potential to markedly change the traditional role of the physician and nurse. In the future, clinicians will oversee the time pattern based analysis and decisions made by the AI to assure that the patients under their care are safe from the nascent, twenty-first century dangers of statistical insignificance and heterogeneous treatment effects. However, this requires that AI systems for acute care provide detailed communication of the factors defining the decisions made by the AI.

To assure that physicians are ready to assume the role of AI oversight, thought leaders should promptly prepare by transitioning the focus of medical education from twentieth century threshold decision making, to twenty-first century time pattern recognition. Potential steps for this transition include;

1. development of teaching archives of time pattern phenotypes for each adverse condition,
2. a renewed focus on the pathophysiology which generates clinical time patterns,
3. development of a time pattern based ontology with communication standards defining the clinical outputs (the handoffs) of AI based analysis, diagnosis and care, and
4. research focused on computer to human handoffs in clinical environments.

## Abbreviations

AI: Artificial Intelligence
HTE: Heterogeneous Treatment Effect
HTM: Human Time Matrix
MRSA: Methicillin-resistant *Staphylococcus aureus*
RCT: Randomized Controlled Trials
RTP: Relational Time Patterns
